# Neuropsychological Heterogeneity in Preschool ADHD: Investigating the Interplay between Cognitive, Affective and Motivation-Based Forms of Regulation

**DOI:** 10.1007/s10802-014-9942-1

**Published:** 2014-09-21

**Authors:** Douglas Sjöwall, Anna Backman, Lisa B. Thorell

**Affiliations:** 1 Department of Clinical Neuroscience, Karolinska Institutet, Stockholm, Sweden; 2 Stockholm Brain Institute, Karolinska Institutet, Stockholm, Sweden; 3 Stockholm ADHD-Center, Stockholm, Sweden; 4 Division of Psychology, Department of Clinical Neuroscience, Karolinska Institutet, Nobels Väg 9, 171 65 Solna, Sweden

**Keywords:** ADHD, Executive functions, Delay aversion, Emotion regulation and preschool

## Abstract

There is a trend toward diagnosing ADHD prior to school entry. Despite this, there is a lack of studies investigating ADHD in the preschool years, at least studies including a large range of different neuropsychological functions. Our knowledge of the independent effects of different neuropsychological functions in relation to preschool ADHD is therefore limited. In order to address this issue, the present study investigated cognitive, affective, and motivation-based regulation in relation to ADHD symptoms in 104 preschool children (age *M* = 67.33 months, *SD* = 10.10; 65 % boys). Results showed that these regulatory processes were all significantly related to ADHD symptoms and that most of these relations remained after controlling for comorbid conduct problems. Most previous preschool studies have only included cognitive regulation, and to some extent motivation-based regulation. By also including affective regulation, we were able to explain a larger proportion of the variance in ADHD symptoms. However, it should be noted that the amount of variance explained was still small in comparison with what has been found in previous studies of school-aged children. This finding could be taken as an indication that further studies examining the nature of preschool ADHD are needed, and that it may be necessary to look beyond the neuropsychological factors that have been linked to the disorder in older children and adults.

Attention deficit/hyperactivity disorder (ADHD) is often diagnosed in middle childhood, but there is a trend toward diagnosing children prior to school entry (see Egger, Kondo, and Angold 2006 for a review). One reason for earlier diagnosis may be research findings showing that preschool ADHD is a serious condition that is linked to severe negative outcomes both concurrently and longitudinally (e.g., Sonuga-Barke, Thompson, Abikoff, Klein, and Brotman 2006). It is therefore important that we gain more knowledge about the neuropsychological underpinnings of the disorder and, thereby, become better able to identify children at risk. One possible benefit of preschool identification is that early intervention could moderate the course of the disorder, and perhaps do so more efficiently before strong behavioral habits have been formed and before the disorder has resulted in secondary deficits (see Sonuga-Barke, and Halperin 2010 for a review). Despite this, there is a lack of studies investigating ADHD in the preschool years, at least studies including a large range of different neuropsychological functions. Cognitive, affective, and motivation-based forms of regulation have been shown to be inter-related, and have all been linked to ADHD in previous research on school-aged children (Nigg, Willcutt, Doyle, and Sonuga-Barke 2005; Shaw et al. 2014; Sjöwall, Roth, Lindqvist, and Thorell 2013). However, the interplay between these three forms of self-regulation in relation to preschool ADHD has not been investigated, and this was therefore the aim of the present study.

## ADHD as a Neuropsychologically Heterogeneous Disorder

Previous research has clearly demonstrated that ADHD involves deficits in multiple neuropsychological functions, such as executive functioning (Barkley 1997) and reaction time variability (e.g., Castellanos, et al. 2005) as well as motivation-based forms of regulation, such as delay aversion (i.e., the tendency to choose a smaller immediate reward rather than wait for a larger delayed reward; Sonuga-Barke 2002). These deficits have been shown to be partly overlapping, but there is also research indicating that they explain unique variance in ADHD (e.g., Sjöwall et al. 2013; Solanto et al. 2001). It has therefore been argued that ADHD is best described as a heterogeneous disorder involving several different neuropsychological pathways (e.g., Nigg et al. 2005).

It is important to emphasize that the notion of ADHD as a heterogeneous disorder is primarily based on the results of studies on school-aged children. However, two meta-analyses on preschoolers have recently been presented. Schoemaker et al. (2012) conducted a meta-analysis of executive functioning in preschool children with ADHD and found a medium effect size for inhibition and small effect sizes for working memory and cognitive flexibility. Another meta-analysis, studying a broader range of neuropsychological deficits in relation to ADHD, found a similar pattern of results for executive functioning, but also a large effect size for delay aversion and a medium effect size for vigilance/arousal (Pauli-Pott and Becker 2011). Interestingly, both meta-analyses showed that most deficits interacted with age such that associations to ADHD were stronger for older compared to younger participants. However, the reverse pattern was true for delay aversion.

With regard to independent effects of different neuropsychological functions in relation to ADHD, very few previous studies have examined this issue among preschool children. However, Thorell and Wåhlstedt (2006) found an independent effect of inhibition, but not of working memory or fluency, in relation to preschool ADHD symptoms. In addition, Skogan et al. (2014) showed independent effects of both inhibition and working memory in relation to ADHD symptoms in their sample of 3 years olds, but effects sizes were very small (r*s* ranging from 0.06 to 0.17) and effects were significant mainly due to the large sample size (*n* = 1,045). To our knowledge, only two previous studies examining independent effects have included motivation-based regulation. In one study, independent effects of cognitive and motivation-based regulation were found in relation to ADHD symptoms (Sonuga-Barke, Dalen, and Remington 2003). However, another study showed an effect only for motivation-based regulation and not for cognitive/motor regulation in relation to ADHD symptoms when using a latent variable approach (Willoughby et al. 2011. In sum, although ADHD appears to be characterized by neuropsychological heterogeneity both in preschool and school age, the relative impact of each deficit may vary with age, which makes it important to conduct further studies of independent effects in preschool samples.

## ADHD and Affective Regulation Deficits

In addition to deficits in executive functioning, delay aversion and reaction time variability, it has been emphasized that deficient emotion regulation should be considered an important aspect of ADHD (see Martel 2009; Shaw et al. 2014 for reviews). Findings from several studies of school-aged children have shown that poor emotion regulation is related to ADHD (e.g., Anastopoulos et al. 2011; Maedgen and Carlson 2000; Walcott and Landau 2004). In addition, these deficits have been shown to be at least partly independent of deficits in other neuropsychological functions in relation to ADHD (e.g., Berlin, Bohlin, Nyberg, and Janols 2004; Blaskey, Harris, and Nigg 2007; Sjöwall et al. 2013).

Very few extant studies have investigated the link between emotion regulation deficits and ADHD symptoms in preschool children, at least studies taking the effect of cognitive deficits into account. One exception is the study by Martel et al. (2013), which showed that both cognitive and affective regulation were related to ADHD symptoms. They conducted a factor analysis in which all included measures formed one factor labeled “control”, and they therefore did not investigate to what extent affective and cognitive regulation explained overlapping variance in ADHD symptoms. Healey et al. (2011), however, examined both additive and interactive effects of cognitive and affective functioning. First, their results showed that executive functioning deficits and negative emotionality were independently related to ADHD symptoms. Second, it was shown that symptom levels were high, except when the child had both well-functioning executive functions and low levels of negative emotionality. Thus, good executive functioning was protective when negative emotionality was low, but not when negative emotionality was medium or high. Neither of the two above-mentioned studies included measures of motivation-based regulation such as delay aversion, and the measures of affective regulation did not include regulation of positive emotions, which should be regarded as a limitation, as regulation of happiness/exuberance has been shown to be linked to externalizing behavior problems both in a non-clinical preschool sample (Rydell, Berlin, and Bohlin 2003) and a clinical school-aged sample (Sjöwall et al. 2013).

As shown above, studies of school-aged children suggest that affective regulation is related to ADHD symptoms independent of cognitive regulation, but little is known regarding to what extent this finding can also be applied to preschool children. Affective regulation is typically viewed as developing earlier than cognitive regulation due to its reliance on subcortical structures (Nigg and Casey 2005). It has therefore been argued that it may be particularly useful to investigate affective regulation in relation to ADHD in preschool children (Martel 2009). This is in line with the idea that differences between ADHD children and controls are largest when investigating neuropsychological functions that have had a chance to develop sufficiently among normally developing children, but not among children with ADHD as they are developmentally delayed (cf. Barkley 1997).

An important issue with regard to the link between ADHD and emotional functioning is that previous research has often used relatively general measures, which have included both emotion regulation as well as how often and how intensely the child reacts emotionally (i.e., emotional reactivity). Separating these two constructs is difficult. However, a child with infrequent and flat emotional reactions may display poor regulation and an emotional child may be a relatively good regulator. It has therefore been argued that it is important to distinguish emotion regulation from emotional functioning in general in order to better understand which aspect of emotional functioning that is related to different behavior problems in children (e.g., Cole, Martin, and Dennis 2004 for a review).

A final issue of importance when measuring emotion regulation is that some of the rating scales used in previous studies (e.g., Conners Rating Scale) include items that overlap with symptoms of Oppositional Defiant Disorder (ODD) and/or Conduct Disorder (CD). As ADHD and ODD/CD often co-occur (e.g., Waschbusch 2002), it has been argued that it is important to investigate the role of ODD/CD symptoms when examining the relation between affective dysregulation and ADHD (e.g., Martel 2009). A large population based study on preschool children even showed that negative emotionality is not primarily linked to ADHD, but to the combination of ODD and internalizing problems (Stringaris, Maughan, and Goodman 2010). In sum, previous ADHD studies have generally been unclear concerning exactly what aspect of emotional functioning they have investigated. In our opinion, future research should employ measures that are as specific as possible, as this would lead to more in-depth knowledge about the link between affective regulation, ADHD, and comorbid disorders such as ODD and CD.

## Aim of the Present Study

The aim of the present study was to investigate multiple forms of regulation and their independent associations with ADHD symptoms in preschool children. As described above, previous studies have operationalized the construct ‘affective regulation’ in different ways, and the major focus has been on negative emotions. In order to address these limitations, we studied emotion regulation using a questionnaire that focused specifically on the regulatory aspect of emotional functioning and that allowed us to study regulation of specific emotions, including happiness/exuberance. ADHD symptoms were studied using ratings spanning from low to high symptom levels as it has been argued that ADHD is best characterized as a dimension rather than as a discrete category (e.g., Marcus and Barry 2011; Sonuga-Barke and Halperin 2010). This indicates that there is a quantitative rather than a qualitative difference between children diagnosed with ADHD and controls, and that clinical studies need to be complemented with studies examining the full range of symptom severity in order to fully understand the neuropsychological heterogeneity in ADHD. We hypothesized that all three forms of regulation would explain unique variance in ADHD symptoms. With regard to affective regulation, we hypothesized that the regulation of both negative and positive emotions would be significantly related to ADHD symptoms.

## Method

### Participants

The present study included 104 preschool children (36 girls) between 4-6 years of age (see Table 1 for demographic data). In order to obtain a sample of children scoring across the full range of ADHD symptom severity, about 1/3 of the sample was clinically referred. These children had been formally diagnosed with ADHD by a psychiatrist, and the children’s diagnostic status was confirmed at the time of the study using both parent and teacher ratings on the ADHD Rating Scale IV (DuPaul et al. 1998). The five children receiving psychostimulant treatment for ADHD were asked to withdraw medication 24 h prior to testing and all children except one adhered to this. The remaining 2/3 of the sample were typically developing children recruited through local preschools. No exclusion criterion with regard to ADHD symptoms was used for these children, and some children were rated by teachers as having a relatively large number of ADHD symptoms. The total sample is therefore best characterized as spanning the full range of ADHD symptom severity rather than as two discrete groups (i.e., skewness = 0.53 and kurtosis = - 0.76 for inattention; skewness = 0.75 and kurtosis = - 0.56 for hyperactivity/impulsivity, which indicates normality; Kline 1998). The children’s parents provided informed written consent for participation and the local ethics committee approved the study.

**Table 1:** Demographic data

	ADHD (*n* = 37)	Controls (*n* = 67)	Total sample (*n* = 104)
	Mean (SD)	Mean (SD)	Mean (SD)
Age in months	69.89 (9.88)	65.91 (10.01)	67.33 (10.10)
Inattention			
Teachers	1.44 (0.61)	0.53 (0.53)	0.86 (0.71)
Parents	1.99 (0.63)	0.59 (0.41)	1.09 (.84)
Hyperactivity/impulsivity			
Teachers	1.55 (0.85)	0.59 (0.73)	0.95 (0.90)
Parents	2.01 (0.64)	0.56 (0.43)	1.09 (0.87)
Conduct problems	2.24 (0.71)	1.57 (0.64)	1.81 (0.74)
Parental education	2.49 (0.52)	2.64 (0.44)	2.59 (0.47)
Intelligence: block design	22.46 (8.98)	21.13 (8.31)	21.61 (8.54)
	%	%	%
Boys	81	57	65
Parental origin			
Immigrant (mother)	16	20	18
Immigrant (father)	23	22	22

Inattention and hyperactivity/impulsivity = ADHD rating scale IV (DuPaul et al. 1998), Conduct problems = Strengths and Difficulties Questionnaire (SDQ; Goodman 1997), Parental education was measured on a scale ranging from 1 to 3, Intelligence = block design subtest from the WISC-III (Wechsler 1991)

### Measures

The laboratory tests were administered in a separate room at the child’s preschool with the administrator present in the room during the entire procedure. The neuropsychological measures described below were standardized, and some measures were reversed so that high values always indicated poor performance. If more than one measure was available within a domain, the mean value of the different measures was used in the analyses.

#### Cognitive Regulation

Working memory was measured using three tasks. Spatial working memory was measured with the “Find the phone task” (Sjöwall et al. 2013) using the Psytools software (Delosis, London). This task is similar in design to the spatial working memory task included in the Cambridge Neuropsychological Test Automated Battery (CANTAB; Owens et al. 1990). In our version of the task, telephones are shown on the computer screen and the task is to remember which telephone has already rung to avoid selecting the same phone several times. The number of times the children returned to a phone that had already rung was used as a measure of spatial working memory. The Children’s Size-Ordering Task (McInerney, Hrabok, and Kerns 2005) was used to measure verbal working memory. In this task, the administrator reads aloud progressively longer lists of common objects (e.g., pencil, train, ball) and the child is asked to repeat them in order of object size, from smallest to largest. The total number of word pairs organized in the correct order was used to measure verbal working memory (maximum = 42). Verbal working memory was also measured using the backward condition of the digit span subtest (Wechsler 1991). The score used was the total number of correct trials.

Inhibition was measured with two tasks using the Psytools software (Delosis, London). The first task was based on the go/no-go paradigm. The particular version used here was originally developed by Berlin and Bohlin (2002) and consists of pictures depicting a blue square, a blue triangle, a red square, and a red triangle, which are presented on a computer screen. During the first part of the task, the child is instructed to press a key (“go”) when a frequent stimulus appears on the screen, but to make no response (“no-go”) when an infrequent stimulus appears. Altogether the task includes 60 stimuli with a “go-rate” of 77 %. The score derived from this task was number of commission errors (pressing the key when a “no-go” target was presented).

**Fig. 1:**
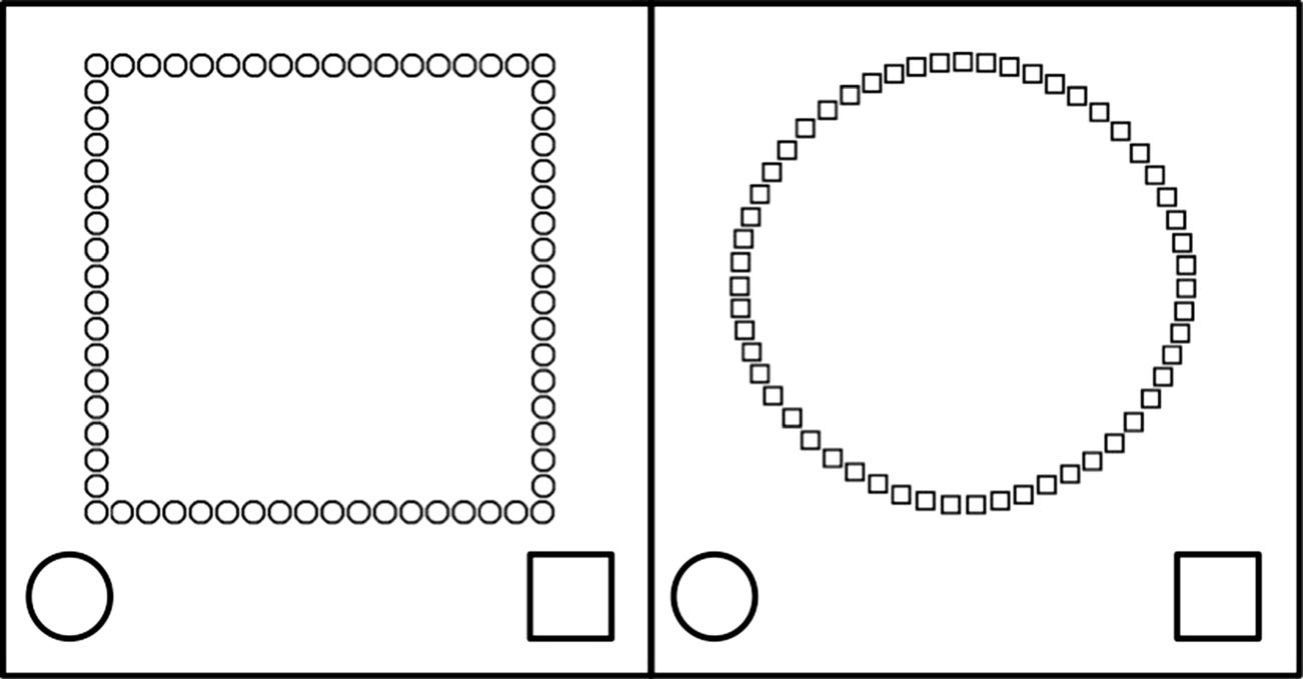
The two stimuli used for the Navon task

The second measure of inhibition was a Navon task (Miyake, Friedman, Emerson, Witzki, and Howerter 2000). In our version, which has been previously used by Sjöwall et al. (2013), a circle consisting of small squares, or the opposite, a square consisting of small circles, was displayed on the computer screen (see Fig. 1), and the participants were instructed to respond to local stimuli in one session (e.g., the small squares making up the circle) and global stimuli in the other (e.g., the circle made up by the squares). Thus, for the stimulus to the left, the correct response would be “square” when asked to respond to global stimuli and “circle” if asked to respond to local stimuli. The children made a response by pressing a key to the left on the keyboard to respond “circle” or a key to the right to respond “square”. In order to make the correct response clear to the children, and to decrease the memory demands of the task, a circle was displayed in the lower left corner of the computer screen and a square in the lower right corner (see Fig. 1). The children were presented with one local and one global session with 20 stimuli in each session. The score used was the total number of errors for the two sessions (maximum = 40).

Shifting was measured using the Navon task (see description above). A third session was performed in which participants were asked to shift between responding to local or global stimuli. A total of 40 trials were presented, and a square and a circle in the lower corners of the computer screen indicated what stimulus to respond to (local trials = small circle/square, global trials = large circle/square). Number of errors during this last session was used to measure shifting.

Reaction time variability was measured using the standard deviation of participants’ reaction times for correct answers on the non-shifting trials in the Navon task and correct answers on the go/no-go task (see descriptions above).

Sustained attention was measured using the go/no-go task described above under the heading “inhibition”. As a measure of sustained attention, we used number of omissions (i.e., failure to respond to a go stimulus) on the go/no-go task.

#### Motivation-Based Regulation

Delay aversion was measured using the Choice Delay Task (Sonuga-Barke et al. 1992). In this task, the child is asked to make 25 choices between an immediate small reward (two seconds for one point) and a delayed large reward (30 s for 2 points). Delay aversion is measured as the number of times the child chooses the small, immediate reward during the final 10 trials. This task has been previously used in, for example, the NIMH Multimodal Treatment Study of ADHD (Solanto et al. 2001).

#### Affective Regulation

Emotion regulation was measured using parent ratings on the Emotion Questionnaire developed by Rydell et al. (2003). In the present study, we included the questions measuring how well the child can self-regulate his/her own emotions. This includes a total of 12 questions related to regulation of anger, fear, sadness and happiness/exuberance. For each emotion, one general and two specific statements are presented (all items are included in an appendix in Rydell et al. 2003). Ratings are made on a scale ranging from 1 (do not agree at all) to 5 (fully agree), with higher values indicating greater problems with emotion regulation. The mean score of the items for each emotion was used in the analyses. This instrument has been shown to have high test-retest reliability and has been validated against both other rating instruments (Rydell et al. 2003) and self-report measures (Rydell, Thorell, and Bohlin 2007).

#### ADHD Symptoms

ADHD-symptoms were measured with the ADHD Rating Scale IV (DuPaul et al. 1998), which includes the 18 symptoms of ADHD as presented in DSM-IV (American Psychiatric Association 1994). Items are rated on a 4-point scale: never or rarely (0), sometimes (1), often (2), or very often (3). The mean scores for symptoms of inattention and hyperactivity/impulsivity were used in the analyses. Teacher ratings were used to assess ADHD symptoms, as parents assessed emotion regulation and we wanted to avoid source bias. Reliability, measured by consistency, was found to be very high for both symptoms of inattention (α = 0.93) and symptoms of hyperactivity/impulsivity (α = 0.96).

#### Covariates

Conduct problems were measured using teacher ratings on the Strengths and Difficulties Questionnaire (SDQ; Goodman 1997). The SDQ is available in over 30 languages (www.sdqinfo.org/) and has been widely used in epidemiological, developmental and clinical research, as well as in routine clinical and educational practice. The conduct problem subscale used in the present study includes questions about temper tantrums, lying, stealing, aggression, bullying, and disobedience. As described in the introduction, it has been argued that the role of symptoms of ODD/CD needs to be taken into account when examining the relation between affective regulation and ADHD (Martel 2009). In the present study, we therefore aimed to address this issue by using the SDQ conduct problem subscale as a covariate when investigating the relation between neuropsychological functioning and ADHD symptoms.

Intelligence was measured using the block design subtest from the WISC-III (Wechsler 1991), a test that has been shown to correlate highly with full-scale IQ (r = 0.93; Groth-Marnat 1997).

### Statistical Analyses

Very little data were missing in the present study: two children did not complete the shifting trial, one child had no ratings for emotion regulation, and four children lacked ratings for ADHD symptoms. First, all data were screened for extreme values (>3 SD) and all outliers (3 for reaction time variability, and 1 for inhibition) were replaced with the value at three SD. To further establish data integrity, we analyzed the detailed computer files generated by the cognitive tasks to make sure that there were no systematic errors in the data (e.g., children making many omissions in a row, pressing the same button over and over again, or switching between right or left button presses). However, no such systematic errors were identified.

In line with the reasoning that ADHD is better captured as a continuous trait rather than as two discrete categories (e.g., Marcus and Barry 2011; Sonuga-Barke and Halperin 2010), and because our sample was normally distributed with regard to ADHD symptoms (see more information under “participants” above), the data were analyzed using a dimensional approach. To study interrelations between cognitive, affective and motivation-based forms of regulation, we computed partial correlation analyses with control for age and sex. These two covariates were included as they were significantly related to several of the predictors, as well as the outcome variables. Next, we investigated how the different forms of regulation were related to ADHD symptoms by conducting partial correlations between the different forms of regulation and the two symptom domains while controlling for age and sex. We also re-ran these analyses controlling for IQ. The reason for not including IQ as a covariate in all analyses was that such procedure has been questioned (e.g., Dennis et al. 2009), as one runs the risk of removing variance that is of relevance for the dependent variable in question (i.e., ADHD). It has therefore been recommended that results are presented both with and without control for IQ, thereby letting the reader make his/her own interpretation of the results (e.g., Barkley 1997).

To investigate independent effects of the three forms of regulation in relation to symptoms of inattention and hyperactivity/impulsivity, we performed hierarchical regression analyses. This way, we were able to study to what extent affective regulation can explain additional variance in preschool ADHD symptoms, beyond the more established cognitive and motivation-based pathways presented in current ADHD theories (e.g., Castellanos, Sonuga-Barke, Milham, and Tannock 2006; Nigg et al. 2005). Finally, interaction effects between all three forms of regulation were investigated using regression analyses, with the main effects being entered in the first step and the interaction effect in the second step. In line with Cohen (1992), correlation coefficients were interpreted as small (*r* = 0.10), medium (*r* = 0.30) or large (*r* = 0.50).

## Results

First, we investigated interrelations between the different forms of regulation that were included in the study. As can be seen in Table 2, correlations between the three executive functions were weak. Sustained attention was significantly correlated with reaction time variability and these two variables were significantly correlated with executive functioning. Delay aversion was significantly correlated with working memory, but not with any other variables. Measures of emotion regulation showed a few significant relations to cognitive regulation, but these were all small in magnitude.

**Table 2:** Intercorrelations between all variables included in the study, controlling for age and sex (two-tailed)

	1	2	3	4	5	6	7	8	9
1. Inhibition									
2. Working memory	0.21*								
3. Shifting	0.14	0.02							
4. Sustained attention	0.36***	0.33***	- 0.31**						
5. Reaction time variability	0.30**	0.28**	-0.20	0.47***					
6. Delay aversion	- 0.03	0.20*	0.08	0.10	-0.00				
7. Regulation of anger	0.11	0.23*	-0.10	0.22*	0.10	0.12			
8. Regulation of fear	0.22*	0.14	0.04	0.22*	0.11	0.03	0.63***		
9. Regulation of sadness	0.14	0.07	-0.05	0.14	0.02	0.06	0.72***	0.65***	
10. Regulation of happiness/exuberance	0.13	0.25**	-0.12	0.24*	0.08	-0.03	0.63***	0.54***	0.55***

* *p* < .05, ** *p* < .01, *** *p* < .001

### Correlations Between Regulation and ADHD Symptoms

Second, we wanted to investigate how the different forms of regulation were related to ADHD-symptoms (see Table 3). Inhibition, working memory, reaction time variability, sustained attention, as well as delay aversion were all significantly related to symptoms of inattention. However, only inhibition and working memory were significantly related to hyperactivity/impulsivity. In addition, all measures of emotion regulation except for regulation of sadness were associated with both inattention and hyperactivity/impulsivity. All significant effects remained when controlling for IQ.

**Table 3:** Cognitive, affective and motivation-based regulation in relation to symptoms of inattention or hyperactivity/impulsivity (one-tailed)

	Inattention	Hyperactivity/impulsivity
Cognitive regulation deficits		
Inhibition	0.258**	0.241*
Working memory	0.428***	0.363***
Shifting	0.055	0.136
Sustained attention	0.268**	0.083
Reaction time variability	0.292**	0.152
Motivation-based regulation deficits		
Delay version	0.261**	0.092
Affective regulation deficits		
Anger	0.417***	0.389***
Sadness	0.166	0.191
Fear	0.334***	0.325***
Happiness/exuberance	0.417***	0.424***

*p < .05, **p < .01, ***p < .01. Cursive numbers indicate relations that changed to non-significance when controlling for symptoms of ODD

### Independent Effects

Third, we examined to what extent measures of affective regulation could contribute significantly to the explained variance in ADHD symptoms beyond the influence of the other forms of regulation. Using hierarchical regression analyses, we entered the two covariates (i.e., age and sex) in Step 1, and all variables that were significantly correlated with the two ADHD dimensions (except emotion regulation) in Step 2. In Step 3, all significant emotion regulation variables were included. As shown in Table 4, the variables entered in Step 2 were significantly associated with inattention. Altogether, they explained 26 % of the variance, with both working memory and delay aversion contributing independently. Adding the emotional regulation variables in Step 3 increased the explained variance to 37 %, and only regulation of happiness/exuberance contributed independently. For symptoms of hyperactivity/impulsivity, 14 % of the variance was explained by the variables entered in Step 2, with an independent contribution only for working memory. Emotion regulation increased the explained variance to 25 %, with none of the variables contributing independently except for a trend towards a significant effect for regulation of happiness/exuberance.

**Table 4:** Regression analyses examining predictors of ADHD symptoms

	ß	R^2^ change
Inattentive symptoms		
Step 1		0.061*
Sex	- 0.143	
Age	- 0.192^+^	
Step 2		0.258***
Inhibition	0.149	
Working memory	0.346**	
Sustained attention	0.042	
Reaction time variability	0.164	
Delay version	0.187*	
Step 3		0.111***
Anger	0.139	
Fear	0.045	
Happiness/exuberance	0.248*	
Hyperactive/impulsive symptoms		
Step 1		0.107**
Sex	- 0.176^+^	
Age	- 0.263**	
Step 2		0.144***
Inhibition	0.179^+^	
Working memory	0.365***	
Step 3		0.111**
Anger	0.140	
Fear	0.043	
Happiness/exuberance	0.232^+^	

^+^ < .10, *p < .05; **p < .01; *** *p* < .001

### Interaction Effects

Fourth, we investigated whether there were any significant interactions between cognitive, affective and motivation-based regulation. A significant interaction effect would indicate that the different neuropsychological deficits combine synergistically (i.e., that the combination of two deficits has an effect on ADHD symptoms that is larger than the sum of its two parts). Of all possible interactions, only the effect of reaction time variability and regulation of happiness/exuberance in relation to inattention reached significance (β = - 0.21, *p* < 0.05). This significant interaction indicated that the combination of high levels of affective regulation of happiness/exuberance and low reaction time variability was associated with especially low levels of inattention. However, it should be noted that this could have been a chance finding due to the very large number of interactions investigated (i.e., 58 interactions altogether).

### The Role of Comorbid Conduct Problems

Finally, we wanted to investigate whether any of the measures of regulation were related to ADHD symptoms mainly due to the large overlap between ADHD symptoms and conduct problems. The results showed that most of the relations remained the same as those presented in Table 3. The exceptions were that there were no effects of inhibition, regulation of fear, or regulation of anger on symptoms of hyperactivity (see cursive numbers in Table 3).

## Discussion

The present study investigated neuropsychological heterogeneity in preschool ADHD by studying cognitive, affective, as well as motivation-based forms of regulation. Results showed that these regulatory processes were all significantly related to ADHD symptoms. Most previous preschool studies have only included cognitive regulation, and to some extent motivation-based regulation. By also including affective regulation, we were able to explain a larger proportion of the variance in ADHD symptoms. However, it should be noted that the amount of variance explained was still small in comparison with what has been found in studies of school-aged children. The implications of these results are discussed below.

### Cognitive Regulation in Relation to Preschool ADHD

In line with previous meta-analyses on different executive functions, our results showed that the effect sizes varied substantially between different functions, with a small effect size being found for the more complex measure of shifting and close to medium effect size for inhibition. The effect size for working memory was somewhat stronger in the present study compared to that found in the previous meta-analyses (i.e., medium compared to small), but well within the range of the studies included in these analyses (Pauli-Pott and Becker 2011; Schoemaker et al. 2012). Furthermore, our study found medium, or close to medium, effect sizes for sustained attention and reaction time variability, which is in line with the effect size reported for vigilance/arousal in the meta-analysis by Pauli-Pott and Becker (2011).

### Motivation-Based Regulation in Relation to Preschool ADHD

The effect size for delay aversion was marginally smaller (i.e., close to medium compared to close to large) than that reported in the meta-analysis by Pauli-Pott and Becker (2011). However, more surprising was our finding that delay aversion was primarily related to inattention rather than to hyperactivity/impulsivity. Few previous studies have investigated separate relations between delay aversion and the two ADHD symptom domains. However, Castellanos et al. (2006) proposed that cool executive functions (e.g. inhibition, working memory and shifting) are primarily associated with inattention, whereas hot executive functions (i.e. delay aversion and decision-making measured by gambling tasks) have a stronger link to hyperactivity/impulsivity. Some empirical support for this notion has also been found in a non-clinical study of kindergarten children (Thorell 2007) and a clinical sample of adolescents (Toplak, Jain, and Tannock 2005). It is difficult to explain the inconsistency between the present study and previous studies with regard to this issue, but one could speculate that the age of the participants or the nature of the sample (clinical versus non-clinical) could be of importance. It has also been shown (e.g., Martel et al. 2013) that relations between neuropsychological functioning and ADHD can vary between symptom domains depending on whether teachers or parents make the ratings, and this may also explain inconsistencies in findings.

With regard to independent effects, both cognitive and motivation-based regulation were shown to have independent effects in relation to symptoms of inattention, which is in line with a previous preschool study (Sonuga-Barke et al. 2003). Further, we found no significant interaction effects of executive functioning and delay aversion in relation to ADHD symptoms. These results imply that these two forms of regulation do not combine synergistically and that they can act alone in producing an effect. This can be taken as further support for the dual-pathway model of ADHD, in which it is stated that these two processes should be regarded as constituting two separate pathways to ADHD (cf. Sonuga-Barke 2002).

### Affective Regulation in Relation to Preschool ADHD

In addition to cognitive and motivation-based regulation, we included measures of emotion regulation. In line with two previous studies on emotional functioning and ADHD in preschool children (Healey et al. 2011; Martel et al. 2013), we found that emotion regulation was significantly associated with symptoms of both inattention and hyperactivity/impulsivity. In contrast to the study by Healey and colleagues, we focused on the regulatory aspect of emotional functioning, whereas they measured negative emotionality. Thus, our findings could be taken as an indication that ADHD symptoms in preschool are not only related to pronounced and often occurring negative emotions, but also specifically to the regulation of both negative and positive emotions. The finding, that significant relations to ADHD symptoms were observed for both negative and positive emotions, is in line with a previous clinical study on school-aged children (Sjöwall et al. 2013), as well as with one study on a non-clinical sample of preschoolers (Rydell et al. 2003), which demonstrated relations between the regulation of positive emotions and externalizing problems in general (i.e., a measure including both ADHD symptoms and conduct problems). Thus, it is important that future research not use measures that only capture the negative aspect of emotion regulation. In order to better understand the importance of regulation of positive emotions, it might be useful to know more about the rating instrument used in the present study. In particular, it should be noted that it does not ask whether the child is often happy or reacts intensely when happy (i.e., emotional reactivity). Instead, the parent is asked to rate the child’s ability to regulate happiness in general as well as in two specific situations (i.e., when the child wins a game/contest and when the child is playing a game that he/she enjoys very much; see Rydell et al. 2003 for the complete questionnaire). We therefore speculate that the difference between adaptive and problematic exuberance lies in the child’s ability to regulate his/her emotions in a socially acceptable manner.

Another important finding of the present study was that significant independent effects of emotion regulation were seen beyond those of cognitive and motivation-based regulation in relation to ADHD symptoms. Thus, affective dysregulation in preschool ADHD is not simply a secondary consequence of deficient cognitive control. Finally, the present study does not find support for interaction effects of cognitive and affective regulation. These results are not in line with Healey et al. (2011), who showed that negative emotionality and cognitive functioning interact in predicting ADHD symptom severity. The reason for this inconsistency may be explained by differences in the type of measures used. As emphasized by Shaw et al. (2014), it is important that future research clearly operationalizes each component of affective regulation, develop consensus measurement techniques, and investigate how these components interact with one another and with ADHD.

### The Role of Comorbid Conduct Problems in Affective Regulation

After controlling for conduct problems, regulation of happiness/exuberance was still significantly related to both ADHD symptom domains, and regulation of both anger and fear were still significantly related to inattention. These results are in line with Sjöwall et al. (2013), who showed that relations between ADHD and emotion regulation remained after controlling for conduct problems in a clinical sample of school-aged children. In addition, Martel et al. (2013) showed that emotion regulation, as measured using the Gift Delay Task, was significantly related to ADHD, but not to ODD in preschoolers. Together, these results show that the link between affective dysregulation and ADHD cannot be explained as a result of the overlap between ADHD and ODD/CD. However, due to the small number of studies in this area of research that have controlled for ODD/CD, replication of these results are needed before any firm conclusions can be drawn.

### The Importance of Neuropsychological Deficits in Preschool ADHD

A limited amount of variance in inattention (26 %) and hyperactivity/impulsivity (14 %) was explained, even though a wide range of cognitive and motivation-based deficits were included. By adding emotion regulation, we could explain additional variance in both inattention (37 %) and hyperactivity/impulsivity (25 %), but still a large amount of variance remains unexplained. The importance of this finding can be discussed both in relation to previous ADHD studies of school-aged children and in relation to current models of heterogeneity.

If we compare our findings with those of older children, we see that effects for both cognitive and affective regulation have generally been larger in school-aged samples (e.g., Berlin et al. 2004; Sjöwall, et al. 2013; Sonuga-Barke et al. 2010). However, we found a relation between ADHD and delay aversion, whereas several studies of school-aged children have failed to show such an effect (e.g., Karalunas, and Huang-Pollock 2011; Solanto et al. 2007). Together, these results are in line with the conclusion of two recent meta-analyses showing that the relation between cognitive regulation and ADHD becomes more pronounced with age, whereas the relative importance of motivation-based regulation shows the reverse pattern (Pauli-Pott and Becker 2011; Schoemaker et al. 2012). One reason for this could be that cognitive regulation has not yet had a chance to develop sufficiently in the preschool years, even among controls. This would indicate that the ability to detect group differences between controls and children with ADHD (i.e., who are thought to show a developmental delay with regard to self-regulation) is more limited in preschool children (cf. Barkley 1997). Another possibility is that ADHD symptoms are not particularly stable over time when assessed in preschool. For example, Lahey and colleagues showed that as many of 45 % of the children with ADHD diagnosed in preschool did not meet the diagnostic criteria at follow-up at age 8 (Lahey, Pelham, Loney, Lee, and Willcutt 2005).

### Future Directions and Practical Implications

Based on the discussion above, we would like to suggest that an important avenue for future research could be to examine to what extent neuropsychological functioning in preschool can help in identifying children who will show stability in ADHD symptoms over time or develop ADHD later on in life. We would also like to suggest that a comprehensive theoretical model of ADHD needs to not only account for the link between neuropsychological deficits and ADHD symptom levels, but also for the extent to which such deficits can explain the functional impairments associated with the disorder. The link between poor executive functioning and academic achievement has been well established in previous ADHD research on school-aged children (e.g., Biederman et al. 2004; Miller and Hinshaw 2010; Rogers, Hwang, Toplak, Weiss, and Tannock 2011), but there is still a need to conduct more person-oriented research that allows for identification of which neuropsychological subtypes in preschool that are most at risk for poor academic achievement over time. In addition, the role of emotion regulation deficits as a significant mediator between ADHD symptoms and social problems has been acknowledged (Sjöwall and Thorell 2014), but seldom examined in preschool samples. However, there is one non-clinical study available, which showed that it was primarily children with a combination of high levels of ADHD symptoms and poor regulation of happiness/exuberance at age 5 who were rejected by their peers at age 9 (Thorell et al. 2014). More research addressing this issue is clearly needed.

Regarding the practical implications of our findings, it should be acknowledged that measures of executive functioning, delay aversion, as well as regulation of emotion may be useful as screeners for ADHD in the preschool age and should be further investigated. Also, these aspects should be considered when developing early intervention and prevention programs for ADHD. In previous research, computerized programs have been shown to strengthen working memory in preschool children (e.g., Thorell et al. 2009). However, due to the neuropsychological heterogeneity in ADHD, programs that target a broader range of regulatory functions might be more appropriate. An example of this is the New Forest Parenting Program, which relies on the parent as the agent-of-change for promoting better regulatory skills in the child (e.g., Sonuga-Barke, Daley, Thompson, Laver-Bradbury, and Weeks 2001; Thompson et al. 2009). We would also like to acknowledge the promising results from another parent program, The Parenting Your Hyperactive Preschooler Program. This 14-week intervention has an especially strong emphasis on strengthening emotion regulation in hyperactive preschoolers and it has been shown to reduce ADHD symptoms and associated behavior in preschool-aged children (Herbert, Harvey, Roberts, Wichowski, and Lugo-Vandelas 2013). The results of the present study could indicate that even larger effects might have been obtained if the program had focused less on negative emotions, and more on enhancing regulatory skills in situations where extreme levels of happiness/exuberance are inappropriate.

### Conclusions and Limitations

In conclusion, the present study has shown that cognitive, affective and motivation-based forms of regulation are all related to ADHD symptoms in preschool and that it is important to study affective regulation in terms of both positive and negative emotions. In relation to current theoretical models of ADHD, these findings clearly provide support for the notion that ADHD is a neuropsychologically heterogeneous disorder involving multiple pathways (cf. Nigg et al. 2005). One limitation of our study was that we only measured affective regulation using parent ratings. We chose this approach in order to reduce source bias, as teachers rated ADHD symptoms. However, it would be of value to also examine affective regulation using laboratory measures (e.g., Carlson and Wang 2007; Martel et al. 2013; Walcott and Landau 2004), as there is a risk that the relation between ADHD and emotion regulation is overestimated when using questionnaires for measuring both the dependent and the independent variable. Another limitation concerns the size of our sample. The present study had the advantage of including multiple forms of regulatory deficits, but due to the large number of factors included, our sample was not large enough to define discrete subgroups with single or multiple deficits. Finally, the fact that we could only explain a relatively small part of the variance in ADHD symptoms may indicate that there are other aspects that need to be taken into consideration if we are to more fully understand the nature of preschool ADHD and be better able to predict long-term outcomes. In our opinion, one of the most important issues for future research is to examine to what extent environmental factors interact with neuropsychological deficits to influence the course of ADHD.
